# Tumor lysis syndrome following ifosfamide monotherapy in metastatic osteosarcoma: a case report and review of the literature

**DOI:** 10.1186/s13256-022-03469-6

**Published:** 2022-06-28

**Authors:** Steven N. Luminais, Xiao T. Chen, Darwin Roman, Brian Ma, Alexander B. Christ, James S. Hu

**Affiliations:** 1 Department of Internal Medicine, Keck School of Medicine of USC, Los Angeles, CA USA; 2 Department of Orthopaedic Surgery, Keck School of Medicine of USC, Los Angeles, CA USA; 3 Department of Oncology, Keck School of Medicine of USC, Los Angeles, CA USA; 4 Department of Pathology, Keck School of Medicine of USC, Los Angeles, CA USA

**Keywords:** Tumor lysis syndrome, Osteosarcoma, Ifosfamide, Hyperuricemia, Hyperkalemia

## Abstract

**Background:**

Tumor lysis syndrome is an oncologic emergency that involves multiple metabolic abnormalities and clinical symptoms such as acute renal failure, cardiac arrhythmias, seizures, and multiorgan failure, and may be fatal if not promptly recognized. Tumor lysis syndrome occurs most often in patients with hematologic malignancies, and relatively few cases have been described in patients with sarcoma.

**Case presentation:**

A 64-year-old male of Asian heritage presented to his primary care physician with a right lower-extremity mass and was ultimately diagnosed with widely metastatic osteosarcoma. He was treated with one cycle of cisplatin and doxorubicin that was complicated by hypervolemia and hypoxic respiratory failure. Given concerns for volume overload, therapy was changed to single-agent, dose-reduced ifosfamide. After receiving one dose of ifosfamide 1 g/m^2^ (1.8 g total) intravenously over 1 hour, the patient developed renal failure, hyperuricemia, hyperkalemia, hyperphosphatemia, and lactic acidosis. The patient ultimately died from severe electrolyte abnormalities associated with tumor lysis syndrome.

**Conclusion:**

This is the first instance of tumor lysis syndrome described in a patient with osteosarcoma undergoing ifosfamide monotherapy. Clinicians must be vigilant in identifying tumor lysis syndrome regardless of the malignancy type or chemotherapy regimen in order to prevent potentially fatal complications.

## Background

Osteosarcoma (OS) is a rare malignancy of mesenchymal lineage that produces bone matrix and related substances. OS primarily presents in the extremities, up to 98% of the time, with 89% of primary OS tumors occurring in the lower extremities [1], though rarely it can present in unexpected locations such as the urinary bladder [2]. Epidemiological studies based on the National Cancer Institute's Surveillance, Epidemiology, and End Results (SEER) program data suggest that the yearly incidence of OS is between two and four cases per million population with peak incidence between ages 5 and 25 years and over 65 years [3, 4]. OS is not known to frequently cause tumor lysis syndrome (TLS), an oncologic emergency that is more commonly associated with hematologic malignancies.

Primary malignancies of bone account for just 0.2% of all cancers in the USA [5], of which 28% and 56% are OS in adults and children, respectively [6], The reported 5-year relative survival rate in patients 60+ years of age is 17%, though 2-year survival rates fall below 10% in the context of distant metastases [3]. The rate of distant metastases is approximately 12.4%, with lungs (86.7%) or other bones (9%) being the most common sites of disease spread [1]. The treatment of unresectable metastatic OS may involve radiation therapy [7] or combination chemotherapy. While there is no consensus on a gold-standard chemotherapy regimen, first-line therapy typically includes cisplatin and doxorubicin [8–10] with or without high-dose methotrexate (MAP regimen) [10–13] and/or ifosfamide [14]. Second-line therapy may include high-dose ifosfamide ± etoposide [15, 16], regorafenib [17], and sorafenib ± everolimus [18, 19].

TLS is a rare constellation of metabolic abnormalities that may occur spontaneously in widely metastatic malignancy or as a sequela of chemotherapy as cells lyse and release their intracellular contents into the surrounding tissue and, eventually, the systemic circulation. The majority of reported TLS cases have occurred in hematologic malignancies with rapid cellular turnover such as Burkitt’s lymphoma and various leukemias [20], though TLS has occasionally been described in mesenchymal-derived tumors. Several chemotherapy agents that have been implicated in TLS include thalidomide, bortezomib, hydroxyurea, paclitaxel, fludarabine, and etoposide [21]. Common laboratory findings include hyperuricemia, hyperkalemia, hyperphosphatemia, lactic acidosis, and hypocalcemia. TLS may result in acute renal failure, cardiac arrhythmias, seizures, multiorgan failure, and, in the most severe cases, death [22]. The mainstays of both TLS prevention and treatment include hydration for renal protection, electrolyte correction, and uric acid management with medications such as rasburicase. Clinically, TLS is a medical emergency that requires prompt recognition and immediate treatment. The current report is the first-reported case of acute TLS following ifosfamide monotherapy in a patient with metastatic intramedullary OS.

## Case presentation

### Prior medical history

The patient is a 64-year-old Asian male with a past medical history of hypertension and hyperlipidemia. The patient had no known family history of malignancy, though he had a personal history of high-risk prostate adenocarcinoma, which was diagnosed 7 years prior to his presentation for OS. This was staged as cT2N0M0 with a Gleason score of 4 + 5 = 9. He was treated with definitive radiation and androgen deprivation therapy (ADT) with leuprolide depot for 2 years. While off ADT, his prostate specific antigen remained less than (nadir + 2) with a nadir of 0.12.

### First hospitalization

**Fig. 1:**
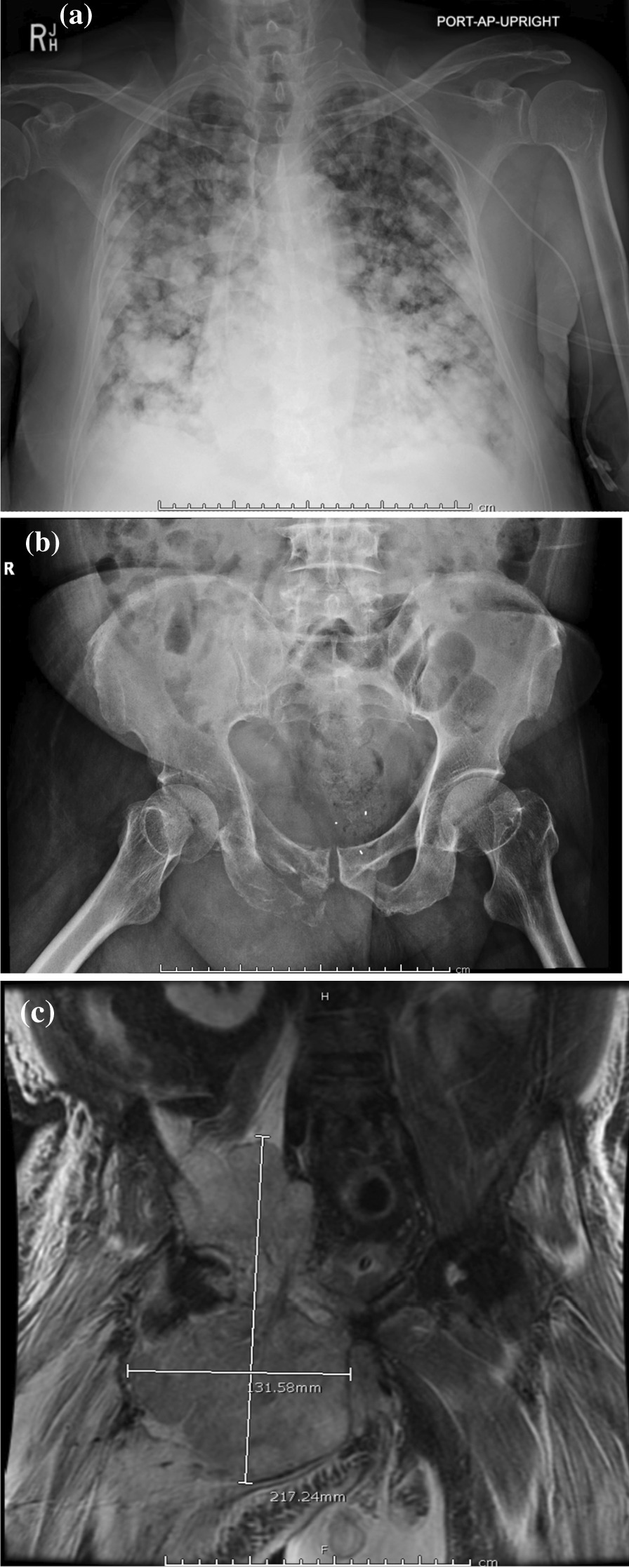
**a** Anterioposterior (AP) chest X-ray demonstrating innumerable pulmonary nodules and masses consistent with metastatic disease. **b** AP pelvis X-ray demonstrating pathological fractures involving the right superior and inferior pubic rami, right acetabulum, and pubic symphysis. **c** Representative coronal MRI cross section demonstrating a large multilobulated, irregular mass involving the right hemipelvis with intraosseous and soft tissue components. The mass demonstrates predominantly low T1 signal and heterogeneous STIR signal measuring (in unshown cross-sections) 15.3 × 21.7 × 12.7 cm. The mass completely replaces the marrow space of the right acetabulum extending into the ilium, pubis involving the pubic symphysis, and ischium with associated pathologic fractures and destruction of the cortex. There is mass effect upon the right iliopsoas musculature with likely invasion. The same process is seen within the left hip rotators and adductors.

**Fig. 2:**
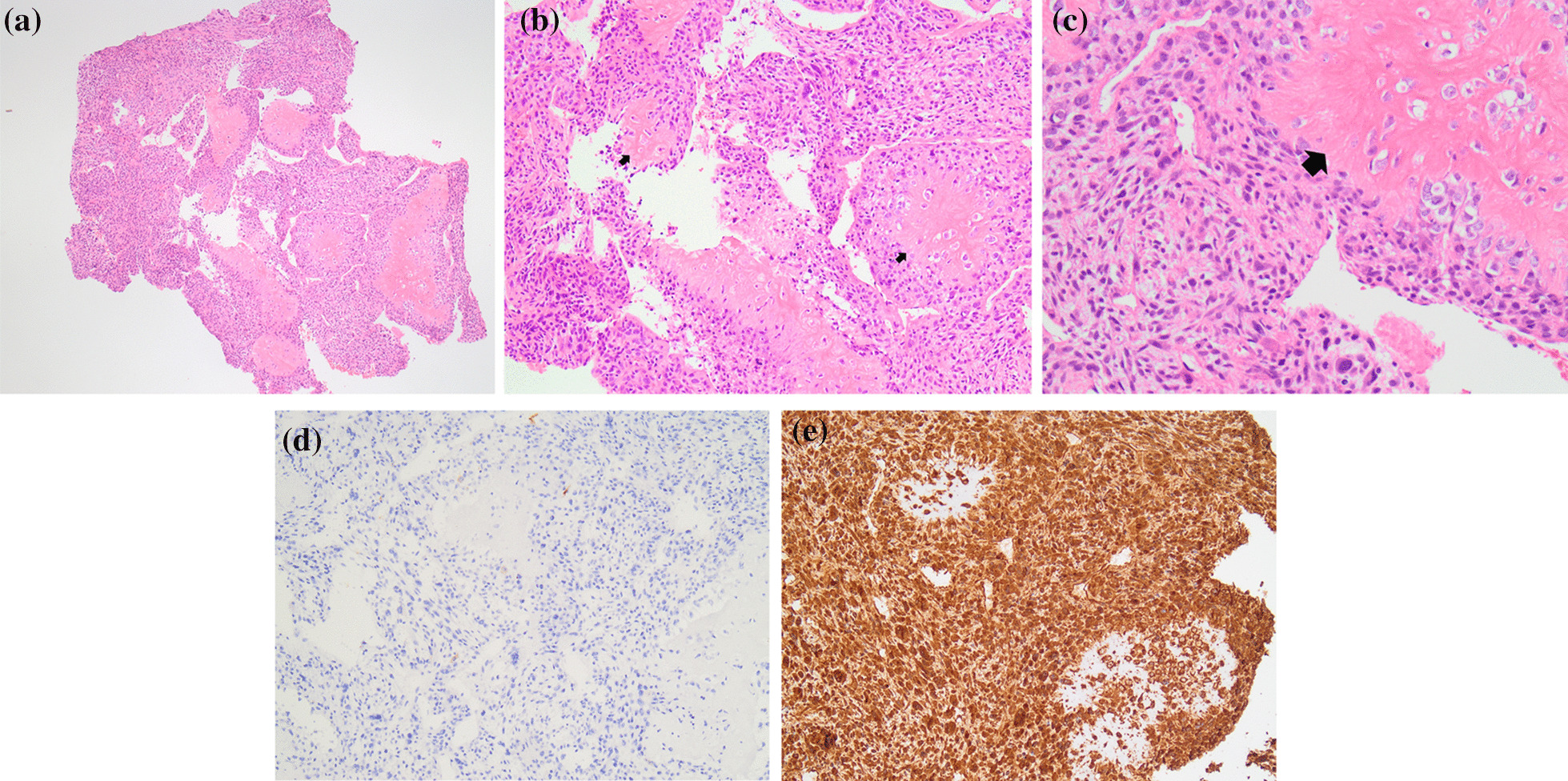
**a**–**e** Right lower-extremity biopsy microscopy showing hematoxylin and eosin (H&E) staining at 4× (**a**), 10× (**b**), and 20× (**c**) magnification showing malignant spindle cell proliferation with areas of osteoid deposition (arrows) all consistent with osteosarcoma. **d** 10× magnification showing negative Ck AE1/AE3 stains, ruling out carcinoma. **e** 10× magnification showing positive vimentin staining highlights the spindle cell portion of the tumor, demonstrating their mesenchymal origin

The patient presented to his primary care physician with a right-sided thigh mass. Before further workup could be completed, the patient presented to the emergency department (ED) with progressive shortness of breath and right lower-extremity edema. In the ED, he was noted to be tachycardic and hypoxic and admitted for further workup. A contrast-enhanced computed tomography (CT) of the chest was negative for pulmonary embolism but positive for innumerable pulmonary metastases up to 4.0 cm in size. A contrast CT and magnetic resonance imaging (MRI) of the abdomen and pelvis demonstrated a large, multilobulated, destructive mass of the superomedial right thigh and pelvis with associated pathologic fractures, as well as multiple hepatic lesions (Fig. 1a–c). A core biopsy of the right lower-extremity soft tissue mass was consistent with high-grade OS and stained positive for vimentin (Fig. 2a–e). The patient’s respiratory symptoms subjectively improved, and he maintained oxygen saturation on 1–2 L of supplemental oxygen; he was discharged home on supplemental oxygen as well as mechanical support for ambulation.

### Second hospitalization

Approximately 1 week later, the patient was seen in an oncology clinic and noted to be tachycardic with 130 beats per minute, respiratory rate of 38 breaths per minute, and hypoxic to 87% on room air. He was admitted that same day for consideration of urgent chemotherapy given the size and number of his pulmonary metastases. CT-guided biopsy of right lung mass was consistent with high-grade OS. Orthopedic evaluation determined he was not a surgical candidate for a hemipelvectomy given the extensive lung disease and oxygen requirements. Systemic chemotherapy was initiated with a planned 28-day cycle of cisplatin (100 mg/m^2^) over 2 hours on day 1 and doxorubicin (25 mg/m^2^) over 4 hours on days 1 through 3. Prior to doxorubicin being started, the patient decompensated requiring additional supplemental oxygen support with high-flow nasal cannula (50 L, 60%). Laboratory results were not consistent with TLS; potassium and phosphorus were within reference ranges and unchanged from prior, while uric acid was slightly elevated (8.5 mg/dL, reference range upper limit of normal 8.2 mg/dL). Repeat CT scan was negative for pulmonary embolism. Given worsening bilateral lower-extremity edema and significant fluid administration with cisplatin, hypervolemia was determined to be the cause of his worsening respiratory status, and the patient was diuresed with intravenous furosemide. He developed a multifactorial acute kidney injury (AKI) (CT contrast, cisplatin), though it resolved over time without hemodialysis. As his respiratory status improved, he received 3 days of doxorubicin therapy to complete cycle 1 of cisplatin/doxorubicin. Ten days after the completion of doxorubicin, the patient was briefly transferred to the MICU for hypotension, while in the ICU he was found to have an extended spectrum beta-lactamase *Escherichia coli* bacteremia that was treated with meropenem. The remainder of his hospital course was uncomplicated, and he was discharged home with home intravenous (IV) antibiotics and oxygen on hospital day (HD) 28.

### Third hospitalization

The patient was readmitted 9 days later for scheduled cycle 2 of cisplatin/doxorubicin systemic treatment. Shortly after the cisplatin and doxorubicin infusions were started on HD 0 (34 days after initial cisplatin dose), he became more hypoxic, requiring bi-level positive airway pressure (BiPAP) support to maintain his saturation. IV fluids and chemotherapy were immediately held, and the patient was upgraded to the progressive care unit (step down). At the time, the patient was clinically volume overloaded with significant bilateral lower-extremity edema. Over the next several days, the patient was diuresed; he continued to require BiPAP support to maintain SpO_2_ ≥ 92%.

Given persistent hypervolemia, the decision was made for a trial of reduced dose ifosfamide (1000 mg/m^2^) monotherapy, with the plan to give daily on days 1 through 5. The patient received his first dose of ifosfamide on HD 7. On HD 8, the patient developed worsening hypoxia and tachypnea. The patient developed worsening metabolic and respiratory acidosis, and the diagnosis of TLS was made. The patient's laboratory values are summarized in Table 1.

**Table 1:** Review of tumor lysis syndrome in sarcomas

Author	Year	Patient age, years	Patient gender	Malignancy	Treatment(s)
Qian *et al.* [23]	2009	44	Male	Retroperitoneal soft tissue sarcoma	Cisplatin Adriamycin Dacarbazine
Gold *et al.* [24]	1993	66	Male	Gastric leiomyosarcoma	Cyclophosphamide Autolymphocyte transfusion
Khan and Broadent [25]	1993	9	Female	Embryonal rhabdomyosarcoma	Carboplatin Epirubicin Vincristine
Ahmed *et al.* [26]	2019	71	Female	Undifferentiated endometrial stromal sarcoma	Paclitaxel Carboplatin
Hiraizumi *et al.* [27]	2011	36	Female	Epithelioid leiomyosarcoma (with focal rhabdomyosarcomatous differentiation)	Vincristine Actinomycin-D Cyclophosphamide
Catania *et al.* [28]	2017	65	Female	Extraskeletal osteosarcoma	None (spontaneous)

The patient was treated with 4 mg of rasburicase, IV furosemide, and intravenous fluids. In accordance with patient and family wishes, the patient was not intubated for respiratory failure and hemodialysis was not offered. Overnight into HD 9, the patient continued to have worsening lactic acidosis despite maximal medical management and noninvasive ventilatory support. The patient’s sinus tachycardia decompensated to asystolic cardiac arrest on HD 9, and he was pronounced deceased.

## Discussion

Prior literature on TLS in OS, and sarcomas in general, is sparse. The available data are limited to case reports, which are summarized in Table 2. While previous case reports describe TLS in multiple different sarcoma types, age groups, and chemotherapy regimens, metastatic spread was present in every patient, indicating that substantial tumor burden likely plays a role in development of TLS, even in mesenchymal tumors [29]. A literature search using PubMed including the terms “osteosarcoma” and “tumor lysis syndrome” yielded a single article by Catania *et al*. describing spontaneous TLS in a patient with metastatic extraskeletal OS [28].

**Table 2:** Patient laboratory values during admission

Laboratory test	First hospitalization (32 days prior to day 1)	Day 1	Day 2	Day 3	Day 4	Day 5	Day 6	Day 7	Day 8 (ifosfamide C1D1)	Day 9	Day 10
Uric acid (mg/dL)	8.5	N/A	N/A	N/A	N/A	N/A	N/A	N/A	N/A	17.1	15.7
Creatinine (mg/dL)	0.67	1.21	1.24	1.63	1.55	1.38	1.24	1.25	1.3	1.66	1.95
Lactate (mmol/L)	2.6	2.3	N/A	2.7	2.8	2.6	2.1	2.3	3.4	6.4	N/A
Sodium (mmol/L)	138	142	144	141	142	141	144	145	150	155	158
Potassium (mmol/L)	4.1	3.7	4.3	4.1	4.1	3.7	3.9	3.8	4.2	4.7	5.3
Phosphorus (mg/dL)	4.5	N/A	5.3	3.5	2.9	2.8	3.5	3.8	4.9	5.6	6.2
Calcium (mg/dL)	8.3	8.6	8.7	7.9	8.2	8.4	8.6	8.9	8.9	8	8.4

Our patient completed only one cycle of first-line cisplatin and doxorubicin therapy during which he required supplemental oxygen and medical management of hypervolemia. He was admitted for scheduled cycle 2 of cisplatin and doxorubicin, though was not able to receive cycle 2 given hypervolemia and hypoxia. After an extensive discussion of risks and benefits with the family, ifosfamide monotherapy at a significant initial dose reduction was given in attempt to induce a partial remission in a patient with a significant decrement in performance status. After one dose of ifosfamide, the patient had clinical and laboratory evidence of TLS, which ultimately led to cardiac arrest. TLS was an unexpected sequela of treatment given the initial 50% ifosfamide dose reduction and a lack of previous literature associating ifosfamide with TLS. Additionally, TLS is a rare occurrence in solid tumors [30] that has been described only a handful of times within patients with sarcoma [23–27] and only once in an individual with OS [28].

The incidence of TLS varies greatly across different cancer types, occurring in over 20% of hematologic cancers [31] while being so rare in sarcomas and other tumor types as to have no reported incidence [30, 32]. Multiple classification systems exist [33–35], though the commonly used standard for identifying TLS is the Cairo–Bishop classification [35], which takes into account a patient’s baseline metabolic panel and categorizes TLS as laboratory TLS (LTLS) or clinical TLS (CTLS). LTLS is defined by two or more of either absolute changes (uric acid ≥ 8.0 mg/dL, potassium ≥ 6.0 mEq/L, phosphorus ≥ 0.5 mg/dL, and/or calcium ≤ 7.0 mg/dL) or expected changes in these laboratory values >25% from baseline within a 24-hour period, 3–7 days after chemotherapy initiation. CTLS is defined by LTLS with any combination of creatinine > 1.5 times the age-adjusted upper limit, seizure, cardiac arrhythmia, or sudden death. Despite the utility of these criteria, the Cairo–Bishop classification is mostly academic. Astute observation of a patient’s clinical status and a thorough understanding of TLS pathophysiology ultimately dictates a physician’s expedient management of suspected metabolic abnormalities.

Treatments for the different components of TLS include volume expansion with intravenous fluids, diuresis with mannitol/furosemide, urinary alkalinization with sodium bicarbonate, uric acid reduction with allopurinol, febuxostat, rasburicase, and renal replacement therapy [20–22, 30, 36]. Despite these lifesaving measures, mortality rates following acute TLS are considerable, ranging from 7% to 51% [37, 38]. In a study of 63 patients by Darmon *et al*., AKI was the greatest predictor of death following TLS in hematologic malignancies. Patients with AKI had significantly higher ICU (31% versus 4%), in-hospital (51% versus 7%), and 6-month (66% versus 21%) mortality compared with those without AKI [38]. Given the severe consequences of delayed TLS recognition and treatment, it may be ideal to risk-stratify patients on the basis of their likelihood of developing TLS on admission. Several predictors of TLS include preadmission renal dysfunction, hyponatremia, metastatic and/or large tumor burden, male sex, splenomegaly, and pretreatment elevations in creatinine, uric acid, and/or lactate dehydrogenase [22, 30]. Unfortunately, both the data on mortality risks and predictors of TLS derive from literature on hematologic malignancies, and more research is needed to determine which factors are useful in the risk stratification of patients with sarcoma.

Catania *et al*. is the only published report of TLS in a patient with OS. However, several key differences exist between this case report and that by Catania *et al*. First, the OS in the study by Catania *et al*. was extraosseous, which is a rare form that accounts for only 4% of OS [39]. Second, the TLS was spontaneous as opposed to after initiation of chemotherapy. Third, the patient underwent hemodialysis and rasburicase infusion therapy, which promptly resolved the patient’s TLS. Similarities between our case reports include the presence of large tumor burden with multiple lung metastases and osteoblastic cells on tumor histology.

The patient in this report had both clinical and laboratory TLS per Cairo–Bishop criteria [35] with increased uric acid and phosphate, the development of an AKI, and a fatal cardiac arrhythmia. This medical emergency was promptly recognized and treated; unfortunately, the patient died despite maximal medical therapy. This report is the first to describe TLS after ifosfamide chemotherapy in metastatic OS, the second report of TLS in OS, and one of just a few reports describing TLS in sarcomas.

## Conclusion

Acute TLS is an oncologic emergency characterized by a distinct combination of laboratory findings including hyperuricemia, hyperkalemia, hyperphosphatemia, and hypocalcemia. TLS has been scarcely reported in the population of patients with sarcoma, and this is the first report of TLS after metastatic osteosarcoma was treated with ifosfamide monotherapy. In this case, delayed presentation and large tumor burden likely played a role in the development of TLS. It is important to promptly recognize and treat this potentially fatal complication regardless of the tumor etiology as delayed management may lead to permanent multiorgan damage, cardiac arrest, and, ultimately, death.

## Data Availability

Data sharing not applicable to this article as no datasets were generated or analyzed during the current study.

## Abbreviations

OS: Osteosarcoma
TLS: Tumor lysis syndrome
ADT: Androgen deprivation therapy
ED: Emergency department
HD: Hospital day
CT: Computed tomography
MRI: Magnetic resonance imaging
BiPAP: Bi-level positive airway pressure
